# Tunable blood oxygenation in the vascular anatomy of a semi-anthropomorphic photoacoustic breast phantom

**DOI:** 10.1117/1.JBO.26.3.036003

**Published:** 2021-03-16

**Authors:** Maura Dantuma, Saskia Kruitwagen, Javier Ortega-Julia, Rutger P. Pompe van Meerdervoort, Srirang Manohar

**Affiliations:** a University of Twente, Multi-Modality Medical Imaging, Techmed Centre, Enschede, The Netherlands; b Medisch Spectrum Twente, Enschede, The Netherlands; c PA Imaging R&D B.V, Enschede, The Netherlands

**Keywords:** breast phantom, quantitative photoacoustics, photoacoustic tomography, Monte Carlo simulations, standardization, breast imaging

## Abstract

**Significance**: Recovering accurate oxygenation estimations in the breast with quantitative photoacoustic tomography (QPAT) is not straightforward. Accurate light fluence models are required, but the unknown ground truth of the breast makes it difficult to validate them. Phantoms are often used for the validation, but most reported phantoms have a simple architecture. Fluence models developed in these simplistic objects are not accurate for application on the complex tissues of the breast.

**Aim**: We present a sophisticated breast phantom platform for photoacoustic (PA) and ultrasound (US) imaging in general, and specifically for QPAT. The breast phantom is semi-anthropomorphic in distribution of optical and acoustic properties and contains wall-less channels with blood.

**Approach**: 3D printing approaches are used to develop the solid 3D breast phantom from custom polyvinyl chloride plastisol formulations and additives for replicating the tissue optical and acoustic properties. A flow circuit was developed to flush the channels with bovine blood with a controlled oxygen saturation level. To showcase the phantom’s functionality, PA measurements were performed on the phantom with two oxygenation levels. Image reconstructions with and without fluence compensation from Monte Carlo simulations were analyzed for the accuracy of oxygen saturation estimations.

**Results**: We present design aspects of the phantom, demonstrate how it is developed, and present its breast-like appearance in PA and US imaging. The oxygen saturations were estimated in two regions of interest with and without using the fluence models. The fluence compensation positively influenced the ${\mathrm{SO}}_{2}$ estimations in all cases and confirmed that highly accurate fluence models are required to minimize estimation errors.

**Conclusions**: This phantom allows studies to be performed in PA in carefully controlled laboratory settings to validate approaches to recover both qualitative and quantitative features sought after in *in-vivo* studies. We believe that testing with phantoms of this complexity can streamline the transition of new PA technologies from the laboratory to studies in the clinic.

## 1 Introduction

Cancerous tissue is often associated with higher density of blood vessels than healthy tissue due to the process of angiogenesis.^1^ Tumor vasculature is also known to be abnormal in architecture and function, characterized by heterogeneity, tortuousity, chaotic branching, and leaky vessel walls.^1^ These characteristics are potential hallmarks to identify the cancer. Imaging techniques, such as contrast-enhanced ultrasound imaging, dynamic contrast-enhanced magnetic resonance imaging (MRI), and positron emission tomography, can visualize tumor vasculature in the clinical setting with the use of contrast agents and are seeing application in cancer diagnosis and treatment monitoring.^2^

Several new breast imaging methods are under investigation and there are strong indications that we might move beyond the traditional imaging tools in the upcoming decade.^3^ Optical imaging techniques are attractive candidates for imaging blood vessels, since they do not require contrast agents, owing to the high optical absorption coefficient of hemoglobin (Hb) in blood.^4^^,^^5^ Another powerful advantage of using light is the marked spectral differences between the optical absorptions of Hb and oxy-hemoglobin (${\mathrm{HbO}}_{2}$) which can be interrogated using different light wavelengths enabling the extraction of blood oxygen saturation as performed in pulse oximetry.^6^ However, volumetric imaging using light suffers from compromised resolution in depth due to light scattering. Photoacoustic (PA) or optoacoustic imaging avoids this problem by employing an emission strategy where acoustic waves, which undergo low scattering, are emitted by blood on absorption of pulsed light. The method has demonstrated high-resolution and high-contrast visualization of tumor vasculature.^7^^–^^9^

PA imaging is based on the PA effect that converts absorbed time-variant optical energy into acoustic waves, via the physical phenomenon of thermoelastic expansion.^10^^,^^11^ During a measurement, the object of interest is illuminated with pulsed laser light, which is scattered in the tissue and specifically absorbed by tissue chromophores. When the laser pulse is short enough for thermal and stress confinement,^12^ the initial PA pressure $p_{0}$ generated is proportional to the absorption coefficient of the chromophore ($\mu_{a}$ in ${\mathrm{cm}}^{-1}$), the amount of light fluence reaching the chromophore (Φ, in $J/{\mathrm{cm}}^{2}$), and the conversion of this absorbed light energy into a temporal volumetric expansion described by Γ, the dimensionless Grüneisen coefficient, as

$$p_{0}(\lambda ,x)=\Gamma (x)\Phi (\lambda ,x)\mu_{a}(\lambda ,x).$$ (1)

This initial pressure build-up relaxes with the propagation of an acoustic wave that can be detected outside the breast with US detectors.^10^^,^^11^^,^^13^ PA signals will be generated in the blood due to the high absorption contrast of Hb, and images of the blood vessels inside the breast can be reconstructed using various algorithms including acoustic backprojection.^14^^–^^17^

Compared to other optical imaging techniques, PA imaging is highly versatile since it is capable of preserving high resolution at imaging depths up to several centimeters.^18^ Breast imaging is one of the most researched applications of PA imaging due to the shortcomings in the currently used imaging modalities.^19^^,^^20^ The breast also lends itself to easy access from all around and comprises tissues that are not highly attenuating for far-red and near-infrared (NIR) light propagation, and neither for US propagation in the medical US frequency regime. Several studies have already applied PA imaging to the breast, and research in this direction continues.^13^

### 1.1 Quantitative Photoacoustic Imaging

Arguably the most interesting application of PA is its potential ability to non-invasively derive the blood oxygen saturation (${\mathrm{SO}}_{2}$), deep in tissue with high resolution, since the parameter is a measure of tissue metabolism, which is known to be dysregulated in cancer.^21^ The ${\mathrm{SO}}_{2}$ relates to the concentrations of Hb ($C_{\mathrm{Hb}}$) and its oxygenated variant ${\mathrm{HbO}}_{2}$ ($C_{{\mathrm{HbO}}_{2}}$) as

$${\mathrm{SO}}_{2}(x)=\frac{C_{{\mathrm{HbO}}_{2}}(x)}{C_{\mathrm{Hb}}(x)+C_{{\mathrm{HbO}}_{2}}(x)}\times 100\%.$$ (2)

Mapping the breast ${\mathrm{SO}}_{2}$ can potentially be a powerful tool to locate a cancer, which is often characterized by hypoxia.^22^ This information can also enable stratification of tumors,^23^^,^^24^ be used for non-invasive monitoring of oxygenation-related responses to treatments,^25^^,^^26^ and help in defining and refining treatment planning since hypoxic regions are known to exhibit resistance to chemotherapy and radiotherapy.^27^^–^^30^

The optical absorption spectra of Hb and ${\mathrm{HbO}}_{2}$ have clear differences,^4^^,^^31^^,^^32^ and the absorption spectrum of blood ($\mu_{a}(\lambda )$) is a concentration-weighted summation of the two:

$$\mu_{a}(\lambda ,x)=\varepsilon_{\mathrm{Hb}}(\lambda )C_{\mathrm{Hb}}(x)+\varepsilon_{{\mathrm{HbO}}_{2}}(\lambda )C_{{\mathrm{HbO}}_{2}}(x),$$ (3)

where $\varepsilon_{\mathrm{Hb}}$ and $\varepsilon_{{\mathrm{HbO}}_{2}}$ are the molar extinction coefficients in ${\mathrm{cm}}^{-1}\;M^{-1}$ for Hb and ${\mathrm{HbO}}_{2}$, respectively. The distinct absorption spectra of the two chromophores allow to back-calculate their concentrations by spectral unmixing the reconstructed PA pressures acquired with two or more optical wavelengths. The concentrations can however only be estimated accurately when the amplitudes of the reconstructed pressures are compensated by the light fluence reaching the absorbers [Eq. (1)]. Several methods have been developed to model the fluence inside tissues to facilitate this so-called fluence compensation, but the wavelength dependency of the tissue optical properties and the unknown morphology of the breast make it a complex problem.^31^^,^^33^

The most used method is based on linear spectral fitting, but this method becomes inaccurate at tissue depths beyond 1 mm.^31^ More accurate methods are more complex and computationally expensive but succeed up to greater imaging depths. Examples are numerical optical transport models based on the radiative transfer equation^34^ or Monte Carlo (MC) simulations.^35^^,^^36^ Addition of diffuse optical tomography measurements to a PA measurement utilizing the same wavelengths can also compensate up to high depths but lacks spatial accuracy.^37^^,^^38^ These, and other methods,^39^ still need to be improved to reduce the error in the fluence estimations made for the deep breast.

### 1.2 Validation of Algorithms

Given the importance of estimating ${\mathrm{SO}}_{2}$ accurately, it is noteworthy that there are only a few instances of rigorous experimental validations of algorithms.^40^^,^^41^ Most of the approaches have only been tested *in silico*, making it unclear what errors can be expected in experiments due to deviations from ideal instrumental characteristics, but also when assumptions made in the theory break down.^33^ Therefore, there is an urgent need to test these algorithms rigorously in controlled experiments before application in *in vivo* studies which are too complex to start with owing to unknown ground truths.^42^^,^^43^

Several test objects with known optical and acoustic properties have been developed to investigate the accuracy of PA reconstruction algorithms and light propagation models.^44^^–^^46^ Examples are oxygen-tunable cubic or cylindrically shaped phantoms with blood-filled channels,^40^^,^^46^^,^^47^ where the former was used to investigate the depth-dependency of ${\mathrm{SO}}_{2}$ estimations. However, most objects in the literature are simplistic and lack the details of spatial distribution of optical and acoustic properties as is expected in the breast. Further, most objects are designed for 2D PA systems or 3D systems with planar illumination, for which the light propagation inside an object with a simple contour and homogeneous optical properties is relatively easy to model. This is not directly translatable to 3D breast imaging systems, since they can possess multiple light sources and the contour of the breast may not be that well defined. These 3D systems are expected to give the most accurate ${\mathrm{SO}}_{2}$ estimations when an accurate fluence model is used, due to larger detection coverage.^13^ Finding a method to accurately validate quantitative photoacoustic tomography (QPAT) on a breast-like object is therefore needed.

In this work, we develop a 3D semi-anthropomorphic phantom in which wall-less vascular channels are present to carry bovine blood whose ${\mathrm{SO}}_{2}$ is tunable. The phantom is endowed with tissue-realistic optical and acoustic properties, and a morphological complexity that approximates a real breast. The blood vessel channels are coupled to a flow loop circuit with an oxygenator and a bovine blood reservoir. The phantom was imaged with US and PA imaging, to demonstrate its usability. Two dual-wavelength PA measurements were performed in the 3D tomographic PAM 2 system,^14^ both with a different blood ${\mathrm{SO}}_{2}$ level. As a proof-of-concept, we recovered the ${\mathrm{SO}}_{2}$ from the PA reconstructions with and without fluence compensation using light fluence maps derived from MC simulations on digital models of the phantom. The improved ${\mathrm{SO}}_{2}$ measures obtained with the accurate light models compared to images uncompensated for fluence demonstrate the challenges for QPAT and the value of such a phantom.

## 2 Materials and Methods

### 2.1 Phantom Production

In previous work, we presented a method to develop a semi-anthropomorphic PA breast phantom from polyvinyl chloride plastisol (PVCP) formulations.^48^^,^^49^ The phantom was fabricated from custom-made PVCP.^50^^,^^51^ The morphology was partially based on MRI-segmented tissue volumes^52^ to give the model a realistic geometry. Blood vessels were mimicked by embedding solid PVCP strands with higher optical absorption than the surrounding. The limitation of this approach is that the optical absorption of the vessels cannot be made to mimic the signature wavelength dependence of blood, with the consequence that the phantom is not appropriate for QPAT studies. With the realization that the distinct optical absorption spectrum of blood and its dependence on oxygen-content is extremely difficult to replicate using a mixture of dyes,^53^ we develop a new approach that allows the use of blood within the semi-anthropomorphic breast phantom. This is achieved by replacing the solid vessel structures from our earlier work with wall-less channels that can be flushed with blood with a controlled ${\mathrm{SO}}_{2}$.

The breast contains a complex vessel network containing vessels with diameters ranging between tens of microns to a few millimeters.^54^ The network including all the small vessels is highly difficult to fabricate without direct 3D-printing or etching of the structures. This is not yet possible due to the lack of suitable materials that can acoustically and optically mimic breast tissue and blood vessels. In addition, vessels smaller than the imaging resolution will anyway be smeared out or lost to the background in the PA image. Therefore, only larger vessels (1.3 mm) are included in this phantom, and the tumor microvascular environment with blood vessels below the spatial resolution-limit of the imaging system is represented as an average higher absorption coefficient in the tumor mass.

The phantom as described in Ref. 49 consists of fat and fibroglandular tissue-mimicking (TMM) PVCP layers, which are made sequentially. These two layers allow for the inclusion of two channel networks: one in the fat layer just below the skin and one in the fibroglandular layer positioned more centrally in the breast. In total, eight channels were included in this prototype, with only two inflow and outflow ports connected to an external flow-circuit. The skin, fat, and fibroglandular tissue-mimicking PVCP materials used in this phantom were made and characterized according to the protocols described in Ref. 49. The experimentally measured optical and acoustic properties can be found in Figs. S2 and S3 in the Supplementary Material. The required steps to create the tumor models, the hollow channels, and connectors to couple the channels to the flow circuit are described here. Photographs recorded during the phantom production process can be found in Fig. S1 in the Supplementary Material.

#### 2.1.1 Tumor models

Two tumor mimics were made by cutting rough spheres with a diameter of 1.5 to 2 cm from a slab of fibroglandular-mimicking PVCP made according to the protocol described in Ref. 49. The only deviation from the protocol was that 0.01 v/v% black plastic coloring was added to the PVCP mixture to increase the optical absorption coefficient by $0.2\;{\mathrm{cm}}^{-1}$ to mimic the tumor’s increased average optical absorption caused by the dense tumor microvasculature.

**Fig. 1:**
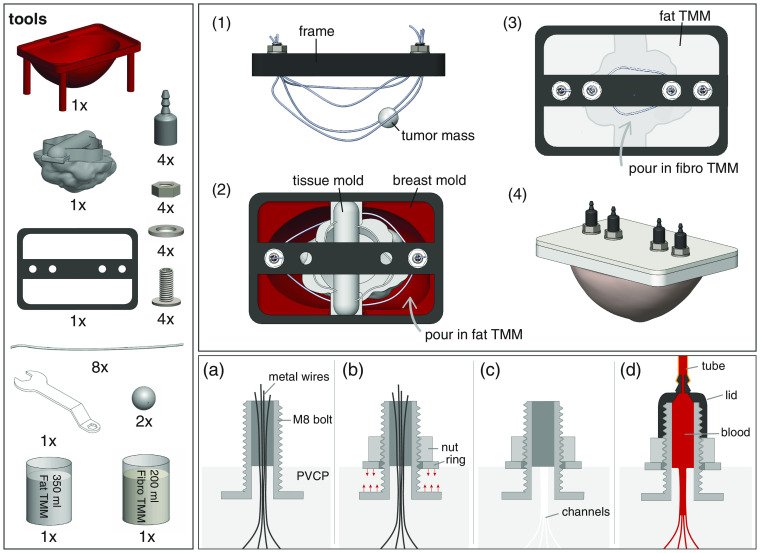
Illustration of the phantom production procedure. The left panel shows all the required tools and the two right panels show steps during the production process, where the lower panel focuses on one of the connectors and shows how the channels can be connected to the flow circuit.

The tumors were pierced with a 1.3–mm-diameter hot metal wire (180°C) to create a channel. To connect this channel to the phantom’s channel network, the tumors were stringed on one of the metal wires that form a channel mold, as described in the next paragraph and shown in Fig. 1(1).

#### 2.1.2 Channels

The high temperature at which the PVCP is poured into the mold (180°C) is a limiting factor in the selection of materials and methods to create the channels. It was decided to make wall-less (or hollow) channels using the advantage of the rigidity and flexibility of PVCP. The method followed was to pour PVCP into the breast mold with embedded metal wires; and pulling the wires out after PVCP solidification, leaving behind smooth channels.

The specific phantom possesses eight hollow channels of which four pass through the fat layer and four through the fibroglandular layer. In both layers, a single tumor is to be present, with one channel passing through the tumor core [Fig. 1(1)]. Four greased 1.3-mm metal wires were arranged as shown in Fig. 1(1). The free-ends of the wires were kept together and fixed inside pre-made connectors that were again positioned on the breast mold with a 3D-printed frame. The wires were bent at different places and positioned inside the volume between the breast surface mold and the fibroglandular tissue mold [Fig. 1(2)]. After pouring in the appropriate PVCP stock for the fat layer and following its solidification, the fibroglandular tissue mold was removed. The process was repeated in the cavity to be filled with fibroglandular-mimicking PVCP [Fig. 1(3)] with a second set of four wires, a tumor mass, and connectors. After solidification of both layers, all metal wires were pulled out one-by-one and the phantom was placed in the pre-made silicone skin sleeve (see Ref. 49 for the skin protocol).

#### 2.1.3 Connectors

Specialized connectors are required to connect the channels inside the flexible PVCP to the tubing of the external flow-circuit. When the blood coming from the tube enters the phantom, it is divided over four smaller channels providing resistance. This requires a solid connector, which can form a tight and reliable seal with the PVCP and which is capable of withstanding high pressures, to prevent the blood to escape via any other routes than the channels inside the phantom.

A customized connector was designed based on a hollowed-out M8 screw, which is partially submerged in the liquid PVCP [Fig. 1(a)]. The hollow connector core allowed the metal wires to pass through, while the connectors were kept in place with the 3D-printed frame that was attached to the breast mold. When the PVCP solidified, the frame was removed and the ring and nut were tightened on the screw to compress the slab of PVCP [Fig. 1(b)], forming a tight seal and preventing blood from leaking along the connector–PVCP interface. After extracting the metal wires one-by-one [Fig. 1(c)], PTFE seal tape was applied to the thread of the connectors and 3D-printed lids were screwed on top to enable the connection to the tubing of the flow circuit [Figs. 1(4) and 1(d)].

#### 2.1.4 Flow circuit and blood saturation regulation

**Fig. 2:**
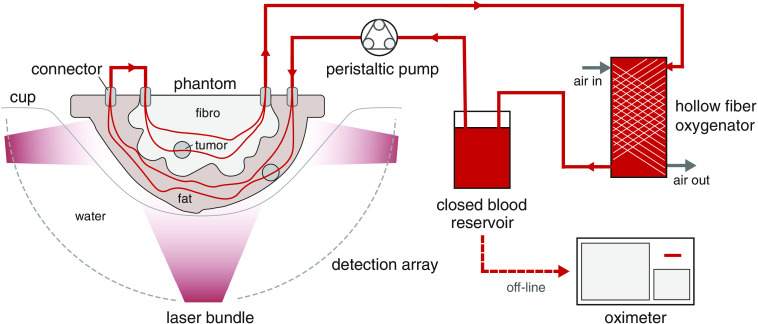
The setup used during the PA measurements. The tunable oxygenation breast phantom is placed in a cup inside the Twente PAM 2 system. A peristaltic pump drives the blood from the reservoir through the circuit, where blood is being oxygenated by a hollow fiber oxygenator. Air flow through this oxygenator can be controlled to regulate the blood oxygenation. SH can be added to the blood reservoir to deoxygenate the blood. Samples are withdrawn from the oxygenator and the oxygenation is measured with the oximeter.

The flow circuit is depicted in Fig. 2. A peristaltic pump (12 V) pumps the blood from an airtight reservoir into the fat layer channel network. The output of this network is connected externally to the input of the second network in the fibroglandular layer (Fig. 2). The output of the second network is connected to tubing to return to the reservoir. Fresh bovine blood containing fibrisol as an anti-coagulant was obtained from a local slaughterhouse one day after butchering and was used within two days. The blood was brought to room temperature before using since it was stored in the fridge at 4°C.

The blood could be oxygenated using a hollow fiber oxygenator (MINIMAX PLUS, Medtronics) connected to instrumental air. Deoxygenation of the blood could be achieved by adding sodium hydrosulfite (SH) powder to the reservoir. Samples of blood were withdrawn to measure the ${\mathrm{SO}}_{2}$ using an off-line commercial oximeter (AVOXimeter 1000E, ITC). After reaching the preferred oxygenation level, the circuit was flushed for about 10 min to stabilize the ${\mathrm{SO}}_{2}$ throughout the circuit. The ${\mathrm{SO}}_{2}$ was re-measured before the start of a PA measurement.

### 2.2 Phantom Validation

#### 2.2.1 Imaging

To verify the US appearance of the phantom, B-mode images were made with the E-cube12a research system (Alpinion Medical Systems, Korea), using the L3-12 probe operating at 6 MHz. The phantom was submerged in water and the channels were filled with air for high acoustic contrast. The phantom was also imaged with commercial CT and MRI machines (see Fig. S5 and S6 in the Supplementary Material).

PA data was acquired using the Twente Photoacoustic Mammoscope 2 (PAM 2) system,^14^ illuminating the breast phantom interleaved at 755 and 1064 nm with 320 and 400 mJ pulse energies, respectively. The laser output of this system is split into two parts, with 50% of the pulse energy illuminating the nipple side of the breast phantom, from the bottom of the tank. The other half was subdivided over 9 fibers to illuminate the phantom from the side. See Ref. 14 for more details about the system. The bottom beam and two of the side beams are illustrated in Fig. 2.

A measurement consisted of a tomographic scan with 45 projections equally divided over 60 deg. The scan involved rotation of the illumination sources and the US detectors arrays around the stationary phantom positioned in a breast supporting cup (size 6) ^55^ in the imaging tank filled with water. The cup was topped-off with water to fill any spaces that may be left between the phantom surface and the cup.

Measurements were performed with two blood ${\mathrm{SO}}_{2}$ levels, being 57.6±1.6% and 8.7±1.6%. Pressure maps were reconstructed for both oxygenation values and for both illumination wavelengths using a backprojection algorithm in an iterative framework^55^ using a reconstruction sound speed of 1496 m/s.

#### 2.2.2 QPAT SO_2_ estimation

We applied three different methods to recover the blood ${\mathrm{SO}}_{2}$ from a tomographic acquisition of the phantom with the sole purpose of demonstrating the value of such a phantom in choosing and optimizing the fluence model. As a first approach, no fluence compensation was used and the ${\mathrm{SO}}_{2}$ was directly calculated from the reconstructed pressures, following Eqs. (1)–(3). For approach (ii), the reconstructed pressures were fluence compensated using a fluence map resulting from MC simulations on a simplified digital model of the phantom using average breast tissue properties assigned to the entire phantom volume. This is an approach that could be applied to *in-vivo* measurements on breasts with unknown internal structures, but with a known breast contour. In approach (iii), the same strategy as in (ii) was used, with the difference that the true ground truth of the phantom was applied, considering the internal phantom fat and fibroglandular morphology.

For the MC simulations, the physical measurement situation of PAM 2 was replicated as best as possible in the MCX toolbox.^56^ The illumination geometry was modeled with the bottom and nine top illumination fibers occupying the 45 rotation steps considered simultaneously (in total 406 sources). The simulation following from this model resulted in one 3D fluence map. Cone-shaped sources were used with directional vectors, apertures, and divergence angles matching the situation in PAM 2 (see Ref. 14 for details). The locations of all sources and the corresponding directional vectors are illustrated in Fig. S4 in the Supplementary Material. The model was checked by comparing the beam geometry from a simulation in a static imaging tank (1 projection) with experimental measurements of the same situation in the PAM 2 system. The digital model of the breast phantom, based on the phantom mold shapes, was incorporated in the MC model. Simulations were performed for both 755 and 1064 nm and the resulting fluence maps were scaled to the laser pulse energies of the respective Alexandrite and Nd:YAG lasers of the PAM 2 system (320 and 400 mJ).

Table 1 lists the optical properties used in the simulations, where the fat and fibroglandular tissue optical properties are the coefficients experimentally measured on the TMM (see Fig. S3 in the Supplementary Material). The values for breast tissue used for approach (ii) are the average of fat and fibroglandular tissues used in approach (iii). The water optical properties were obtained from literature.^57^ The refractive indices were calculated with the Lorenz–Lorenz mixture rule from the PVCP plasticizer refractive indices and their volume fractions,^58^ and the anisotropy factor for the PVCP materials of 0.7 are based on Mie scattering theory as in Ref. 50.

**Table 1:** Experimentally measured optical absorption and scattering coefficients (${\mathrm{cm}}^{-1}$) and the dimensionless anisotropy factors and refractive indices that were used in the MC simulations.

	755 nm		1064 nm		g	n
	$\mu_{a}$	$\mu_{s}^{\prime }$	$\mu_{a}$	$\mu_{s}^{\prime }$		
Water	0.03	$10^{-9}$	0.12	$10^{-9}$	0.95	1.33
Breast	0.44	21.92	0.63	13.88	0.7	1.62
Fat	0.38	25.02	0.56	17.01	0.7	1.49
Fibro	0.49	18.81	0.71	10.74	0.7	1.74

Before fluence compensating the reconstructed pressures, the 3D fluence maps were superimposed on the reconstructions to check and fine-tune their alignment. The ${\mathrm{SO}}_{2}$ was calculated for two regions of interest (ROI), which were selected because they possessed minimal double line reconstruction artifacts and were therefore expected to give the most reliable ${\mathrm{SO}}_{2}$ results. The first ROI contains a vessel that lies in the fat layer, while the second contains a vessel from the deeper fibroglandular tissue layer [Fig. 5(b)]. The used molar extinction coefficients are listed in Table 2.^4^^,^^32^ Here, we assumed that the molar extinction coefficients of bovine blood in the NIR are similar to the ones of human blood as was experimentally proven in Ref. 59.

**Table 2:** The blood molar extinction coefficients (${\mathrm{cm}}^{-1}/M$) for estimating the ${\mathrm{SO}}_{2}$.

	755 nm	1064 nm
$\varepsilon_{\mathrm{Hb}}$	1551.0	134.0
$\varepsilon_{{\mathrm{HbO}}_{2}}$	555.2	867.0

## 3 Results

### 3.1 Imaging

**Fig. 3:**
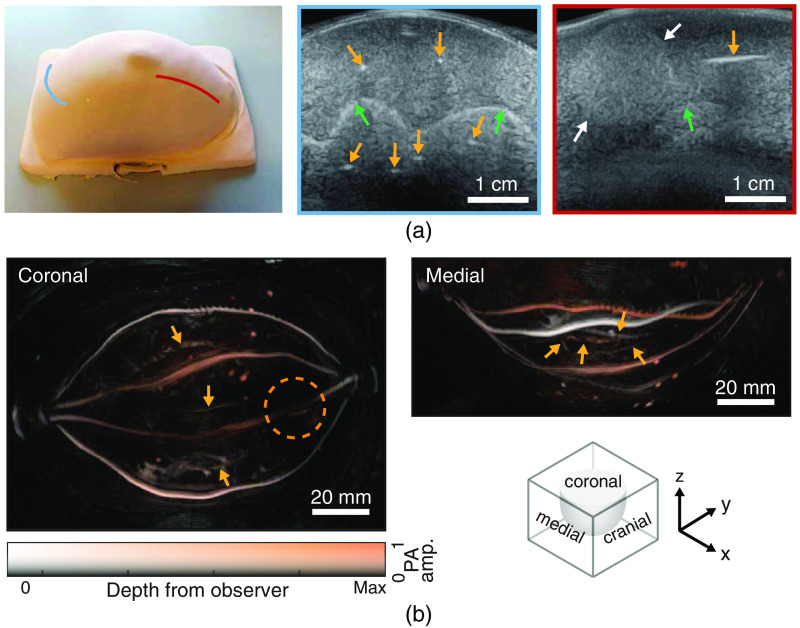
(a) A picture and US images of the phantom acquired perpendicular and parallel to the channel direction. The yellow arrows highlight the channels and the green arrows highlight the interface between the fat and fibroglandular TMM. White arrows point at the tumor. The probe positions on the phantom to acquire these images are marked in red and blue lines. (b) LMIPs along the coronal, medial, and cranial plane of the reconstruction of the PA measurement with 755 nm and a 8.7% ${\mathrm{SO}}_{2}$. The yellow arrows point to the deeper lying vessels in the fibroglandular layer and the circle indicates the location of the shallow tumor.

Images of the phantom obtained with US and PA imaging are presented in Fig. 3. The upper panel shows from left to right, a photograph of the phantom, and two US scans acquired. Each US image is framed with the same color of the line that marks the respective probe position in the photograph. The left US image was acquired perpendicular to, and the US image at right along the channel direction. The images show one of the tumors (white arrow) and the undulating interface (green arrows) between the fat and fibroglandular tissue layers. The air-filled channels (yellow arrows) show up with high echogenicity. Flushing the channels in the phantom with water during US imaging showed that the air is driven out completely and that all vessels can be flushed.

The lower panel shows normalized local maximum intensity projections (LMIPs) along the coronal and medial planes for the PA measurement with 755 nm and a blood oxygenation of 8.7%. The images are color encoded for depth from the observing plane. All channels can be observed (yellow arrows) in the PA reconstructions, with the channels in the fat layer better visible those in the deeper lying fibroglandular layer. At the location of the shallow tumor (encircled), a lower intensity can be observed in the channel, due to a lower light fluence arriving due to tumor “shadowing.”

**Fig. 4:**
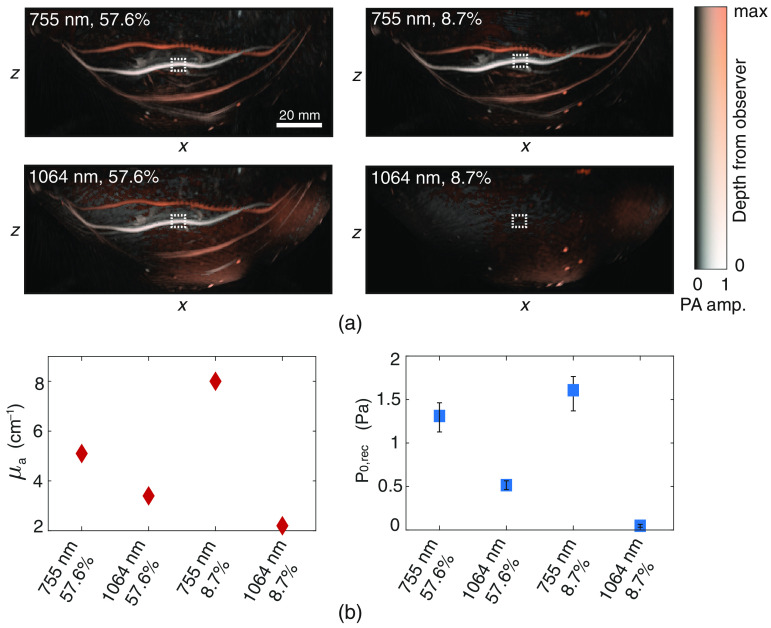
(a) LMIPs of the medial view of the PA measurements with 755 and 1064 nm for the two blood oxygenation levels. (b) The average reconstructed pressures from the vessel pixels in the ROIs highlighted in (a) together with the blood absorption coefficients for the respective wavelengths and oxygen saturations.

The effect of the optical wavelength and the blood saturation values on the PA image is shown in Fig. 4(a). For all four reconstructions, the average reconstructed pressures in the highlighted ROIs are plotted in Fig. 4(b) together with the corresponding absolute blood absorption coefficients. Comparable trends are seen in the pressures and absorption coefficients.

### 3.2 $SO_{2}$ estimations

**Fig. 5:**
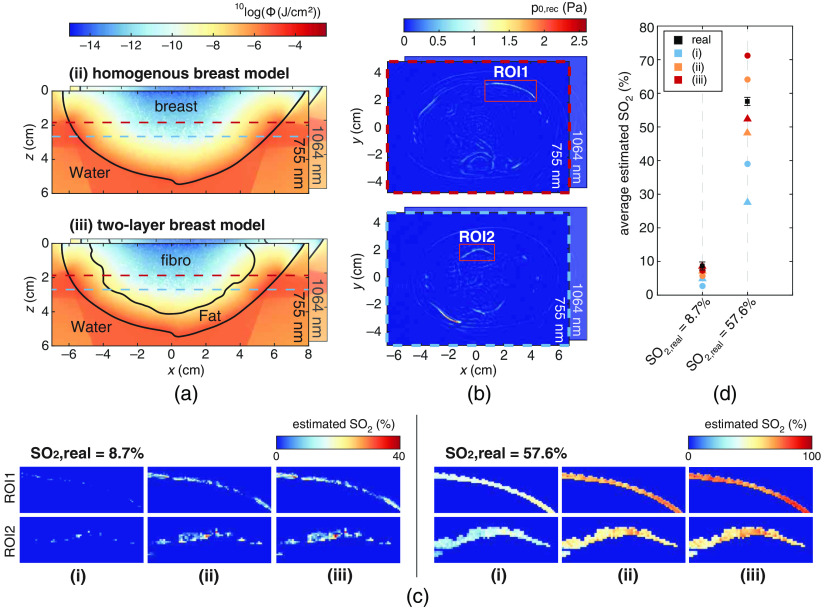
Process to recover ${\mathrm{SO}}_{2}$ values, with (a) the fluence compensation approaches where (ii) and (iii) show the fluence models at y=0, with the contours of the tissue volumes where the MC simulations were performed on. (b) Two slices of the reconstructed pressures of the measurement with the 57.6% ${\mathrm{SO}}_{2}$. The ROIs used for the ${\mathrm{SO}}_{2}$ estimations are highlighted. The MIPs of the final ${\mathrm{SO}}_{2}$ estimations for both ROIs and both measurements are presented in (c) and the found average ${\mathrm{SO}}_{2}$ values of for ROI1 (circles) and ROI2 (triangles) are plotted in (d).

The ${\mathrm{SO}}_{2}$ was calculated in two ROIs (ROI1: 81 v 38×19  px, ROI2: 64×29×14  px) for both PA measurements following the three approaches: (i) no fluence compensation, (ii) fluence compensation based on homogeneous optical properties, and (iii) fluence compensation based on the ground truth two-layer model of optical properties. Figure 5(a) shows slices of the fluence maps at y=0, resulting from the MC simulations for approaches (ii) and (iii). The contours of the tissue volumes used for the two approaches are superimposed on the fluence maps. The reconstructed pressures ($p_{0,\mathrm{rec}}$) at two heights in the phantom (marked by colored dashed lines in A) are shown in Fig. 5(b) boxed by the respective colored dashed lines. Figure 5(c) presents maximum intensity projections (MIPs) of the estimated ${\mathrm{SO}}_{2}$ in the ROIs (marked in red boxes in B) for the two set values of ${\mathrm{SO}}_{2}$ namely 8.7% and 57.6%. The average ${\mathrm{SO}}_{2}$ estimations for both ROIs are plotted in Fig. 5(d). Table 3 also lists the average ${\mathrm{SO}}_{2}$ estimations in both ROIs together with the average absolute deviation from the real ${\mathrm{SO}}_{2}$.

**Table 3:** Average estimated oxygenation values in ROI1 and ROI2 and the average absolute deviation of those from the true ${\mathrm{SO}}_{2}$, for both measurements and for the different fluence compensation approaches.

approach	8.7%			57.6%		
	ROI1	ROI2	average absolute deviation	ROI1	ROI2	average absolute deviation
i	2.7%	4.9%	4.9%	39.0%	27.6%	24.3%
ii	5.7%	7.1%	2.6%	64.1%	48.4%	8.0%
iii	7.3%	8.5%	0.8%	71.2%	52.4%	9.4%

For both PA measurements, approach (i) performs the worst. Approaches (ii) and (iii) clearly perform better and decrease the average absolute deviation from 4.9% to 2.6% and 0.8%, respectively, for the ${\mathrm{SO}}_{2}=8.7\%$ measurement. For the ${\mathrm{SO}}_{2}=57.6\%$ measurement, they significantly reduced the average absolute deviation from 24.3% to 8.0% and 9.4%, respectively. This demonstrates that knowing the breast contour and approximating the breast optical properties significantly increases the accuracy of the ${\mathrm{SO}}_{2}$ estimation. The errors made in the 57.6% measurement are larger than for the 8.7% measurement. Recovering an ${\mathrm{SO}}_{2}$ of 57.6% is more difficult than the 8.7% since the difference in optical absorption between the two wavelengths for the former is significantly lower than for the latter ($1.7\;{\mathrm{cm}}^{-1}$ versus $5.9\;{\mathrm{cm}}^{-1}$).

## 4 Discussion

This work presents for the first time a strategy to develop and use a complex PA breast phantom with tunable blood oxygenation while being semi-anthropomorphic in distribution of optical and acoustic properties. In the phantom that we developed, the optical and acoustic properties of all the tissue mimics of the semi-realistic breast morphology are characterized separately. Bovine blood can be flushed through wall-less channels in the phantom, with a controlled ${\mathrm{SO}}_{2}$. Due to the heterogeneous build-up of this phantom, as in the breast, acoustic waves will be reflected and refracted, which can be challenging for the backward acoustic solvers and induce errors in the ${\mathrm{SO}}_{2}$ estimations. Similar effects hold for the optical propagation. This advanced phantom platform therefore represents an experimental ground truth to test, optimize, and ultimately validate various experimental and algorithmic approaches for performing quantitative ${\mathrm{SO}}_{2}$ estimations in the breast.^31^^,^^39^

### 4.1 Phantom Design

The main body of the phantom is made from fat- and fibroglandular-mimicking PVCP prepared according to the protocols in Ref. 49, with sound speeds, acoustic attenuation, and optical properties mimicking the tissue types. The phantom was given a realistic morphology by using 3D-printed molds. Two wall-less channel networks, both consisting of four 1.3-mm-diameter channels were embedded in the phantom, allowing to be flushed with bovine blood. Two tumor masses with an average higher optical absorption were embedded in the phantom, with each one’s core also being traversed by one of the channels. A novel connector was designed to generally couple a channel in a soft phantom to external tubing while ensuring a good seal. Specifically, in our phantom, the connectors were used to couple the inner channels to an external flow loop system so that bovine blood could be pumped into the phantom without leaks. The connectors have been made such that they can be removed and re-installed, which can also allow MRI or x-ray CT imaging of the phantom without interference from the metal in the connectors.

US B-scans showed that all the channels and tissue layers can clearly be discriminated by looking at their speckle contrast in US reflection mode imaging. Reconstructions of PA measurements on the phantom filled with blood showed expected signatures from all vessels.

An unsolved problem in the phantom design is the skin TMM that detaches from the PVCP when the phantom is submerged in water. This is not a problem for B-mode imaging, since the skin TMM will be pressed against the PVCP with the probe, but does create artifacts in tomographic imaging. A simple solution is to seal the skin layer with PVCP.

In the tumor masses, the expected higher density of microvessels is simplified into an increased overall optical absorption. Since this phantom was designed for 3D PA imaging, where systems have reported imaging resolutions between 0.3 and 1 mm,^13^^,^^14^ individual capillaries (5 to 40  μm^60^) cannot be resolved with these systems. Therefore, we believe that the developed tumor model is a good first approximation and that it serves the current goal. For future work, it could be interesting to develop more advanced tumor models that contain a few chaotically shaped feeding vessels or dilated capillaries. To model the tumor’s increased metabolism, it would be useful to locally and in a controllable fashion deplete oxygen from the blood. This can make the phantom appealing for real-time qPAT studies.

The material optical and acoustic properties of the PVCP TMM were measured and were generally found to be comparable to the measured properties in Ref. 49, which correspond well to fat and fibroglandular breast tissues. The optical scattering coefficient of the fat TMM was however significantly higher, presumably caused by a longer mixing time of the optical scatterers (titanium dioxide) through the PVCP mixture. This resulted in a $\mu_{s}^{\prime }$ that is higher than that of breast fat tissue. The optical absorption of the tumor models was increased with $0.12\pm 0.03\;{\mathrm{cm}}^{-1}$.

### 4.2 QPAT Validation

Measurements with two different wavelengths on two different blood oxygenation levels were performed. The blood ${\mathrm{SO}}_{2}$ values used in this study are low compared to values expected in a real breast and were only meant for demonstration purposes. The PA reconstructions for the two wavelengths and ${\mathrm{SO}}_{2}$ showed clear differences in PA intensity. Blood with a ${\mathrm{SO}}_{2}$ of 8.7% was almost invisible at 1064 nm, due to the low optical absorption coefficient of blood in combination with a higher optical absorption of the breast TMM and water.

PA measurements were performed with the PAM 2 system, which synthesizes a hemispherical recording aperture by scanning curved US detectors arrays around the breast-shaped phantom.^14^ This system, while producing high-resolution and high contrast blood-rich images from the breast, is not directly appropriate for QPAT studies without more complex corrections. The reason is that, while the dominant light incidence on the nipple-side of the phantom remains steady during the acquisition, the nine light injection points higher up the phantom move along with US arrays. This changing of a part of the illumination on the breast phantom during the acquisition is challenging to model in the simulations so as to be exactly the same as in the experiment and can give rise to error. Under the caveat that the acquisition of data with the PAM 2 system is not highly conducive for QPAT, with the goal of demonstrating the utility of such a phantom, the measurements were conducted. Three approaches were applied to recover the ${\mathrm{SO}}_{2}$ in two ROIs within the phantom. The use of no fluence compensation showed average absolute deviations in the recovered ${\mathrm{SO}}_{2}$ from the true ${\mathrm{SO}}_{2}$ of 4.9% and 24.3% for the 8.7% and 57.6% ${\mathrm{SO}}_{2}$ measurements, respectively. MC simulations on approximations of the phantom, considering a homogeneous breast model or a true ground truth of the model both improved the estimation accuracy significantly. The average absolute deviations were decreased to 2.6% and 8.0% for the homogeneous breast model and to 0.8% and 9.4% for simulations on the true ground truth. The remaining differences in the estimation errors between approach (ii) and (iii) indicate that these fluence models have to be highly accurate to recover the true ${\mathrm{SO}}_{2}$ and that the fluence models used here still lack some accuracy, even though the exact ground truth of the optical property distribution is known.

The accuracy in the estimations, in addition to the inherent error in acquisitions of the projections aforementioned, is affected by contributions from: (1) errors in the experimentally determined optical properties of the breast TMMs, (2) errors in the oximeter measurement, (3) inaccuracies in the PA reconstruction, and (4) errors in the inverse optical model. The contributions of (1) and (2) are expected to be small, as the oximeter and the IAD program to measure the TMM optical properties were calibrated. A sound-speed map for (3) and more advanced light models in (4) are expected to have the largest benefit.

The flow circuit used in this study enables to flush the blood through the channels, and the ${\mathrm{SO}}_{2}$ can be increased or decreased by addition of air or SH. Since the blood–air contact is minimized after the preferred ${\mathrm{SO}}_{2}$ is achieved, the ${\mathrm{SO}}_{2}$ remains stable for at least 6 min. A more advanced flow-system, which contains an in-line spectrometer that can provide real-time ${\mathrm{SO}}_{2}$ feedback, could be used for future studies. Examples of these flow systems have already been reported in Refs. 46 and 47.

## 5 Conclusions

We have presented an approach for the development of 3D semi-anthropomorphic PA breast phantoms containing wall-less channels that can be flushed with blood using an external flow-loop. The blood can be saturated with oxygen to various levels using an oxygenator. This phantom allows studies to be performed in PAs in carefully controlled laboratory settings to validate approaches to recover both qualitative and quantitative features sought after in *in-vivo* studies.

PA measurements were performed on the phantom when it was flushed with bovine blood with two different oxygen saturation levels. Image reconstructions without and with fluence compensation were performed and the results analyzed to extract the set values of oxygen saturation. This showed the utility of the phantom and reiterated the importance of accurate light models. The use of such a sophisticated phantom can also present insights into the general imaging performance of imaging instrumentation and recommend specific areas for optimization.

The phantom is also intended for use in dedicated US computed tomography systems (USCT) or hybrid imagers combining USCT and PAT. In such a combined imager, US transmission and/or reflection measurements could provide the breast contour (in cases this is not known from a supporting cup as in Ref. 55), sound speed maps, and attenuation maps, which could be used to improve PAT reconstruction. The breast contour can help to improve the light modeling, and the US transmission parameters could improve the accuracy of the acoustic inversion problem. The breast phantom described can also be used to validate the performance of diffuse optical tomography. Further, for another track of research, PA or US contrast agents could be added to the blood, permitting studies with PA and US Doppler and flowmetry imaging.

## Code, Data, and Materials Availability

All the data and metadata from the measurements described in the paper and supplementary materials will be made available on request for testing of image reconstruction algorithms. The same holds for all the 3D models required for phantom reproduction.
