# Supplementary material for: Tunable blood oxygenation in the vascular anatomy of a semi-anthropomorphic photoacoustic breast phantom
Source: J Biomed Opt. 2021 Mar 16;26(3):036003. doi: 10.1117/1.JBO.26.3.036003 (PMC7961914; doi:10.1117/1.JBO.26.3.036003)
Supplement: Supplementary file 1 [file JBO_026_036003_SD001.pdf]

# Supplementary materials

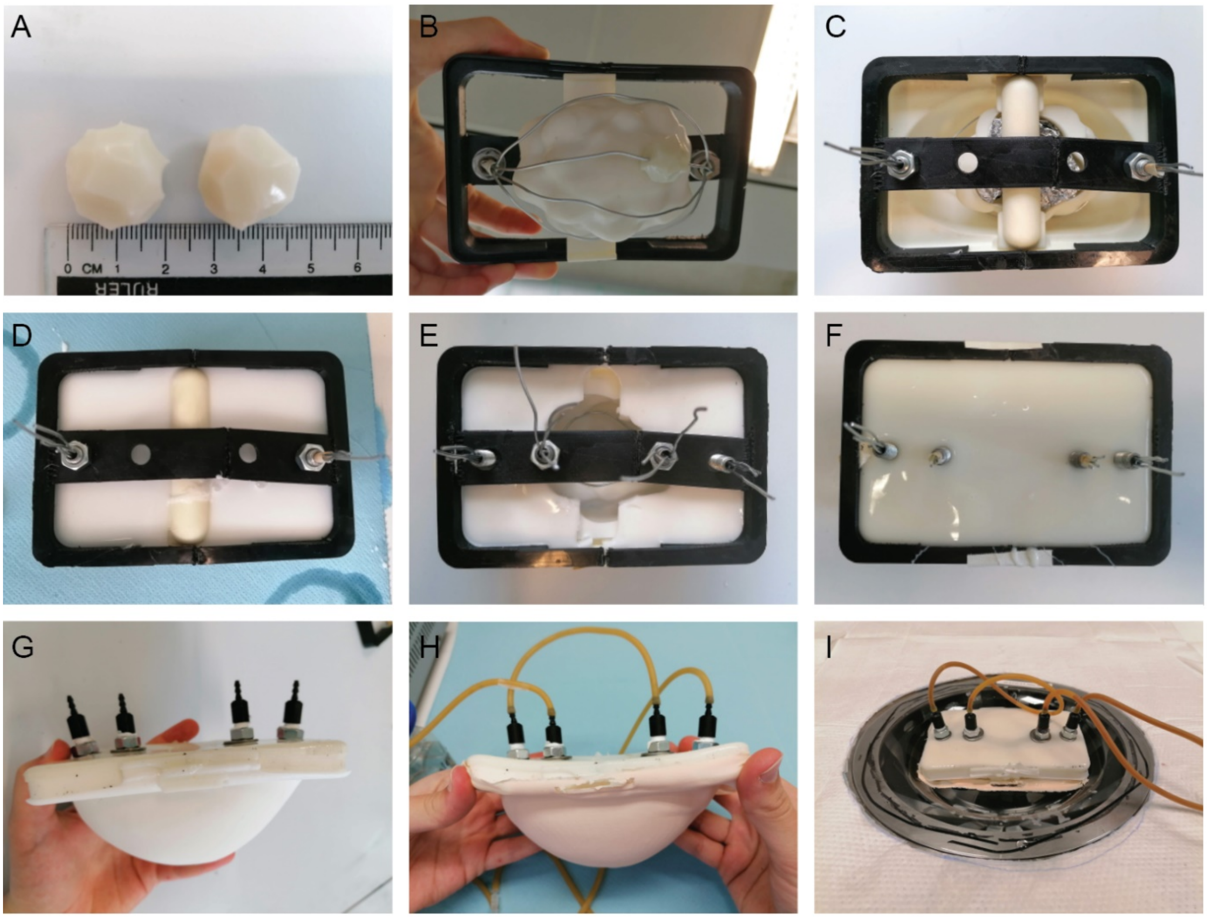


**Figure S1:** Pictures taken during the phantom creation process. (A) Tumour masses. (B) 3D-printed frame with the tissue mould and the wires with one of the tumour masses before placing it in the breast mould. (C) Status after placing it in the breast mould. (D) Result after pouring the fat TMM. (E) Tissue mould removed and the metal wires for the inner channel network are in place. (F) The result after pouring the fibroglandular TMM. (G) The phantom taken out of the breast mould and the 3D-printed lids screwed onto the connectors. (H) The phantom placed in the pre-made skin and connected to the tubing of the flow-circuit. (I) Picture of the phantom in the PAM2 system.

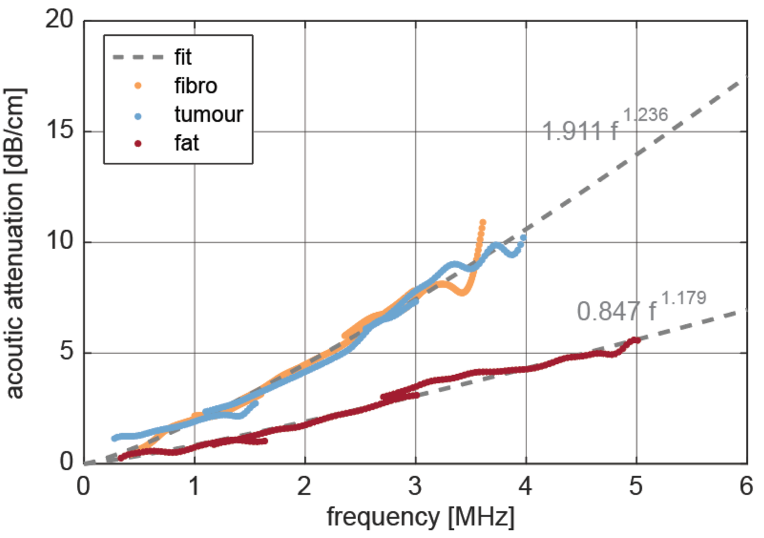


**Figure S2:** Acoustic attenuation spectrum for the fibroglandular, tumour and fat mimicking PVCP measured with 1, 2.25 and 5 MHz transducers. The dashed lines show the power-law fits for the fat and fibroglandular TMM.

**Table S1:** Sound speeds of the TMM at 22°C. The sound speeds are the average with the variation ranges from three transmission measurements with 1, 2.25 and 5 MHz single-element transducers.

| TMM | sound speed [m/s] |
|---|---|
| fat | 1406 ± 2 |
| fibro | 1512 ± 1 |
| tumour | 1524 ± 3 |

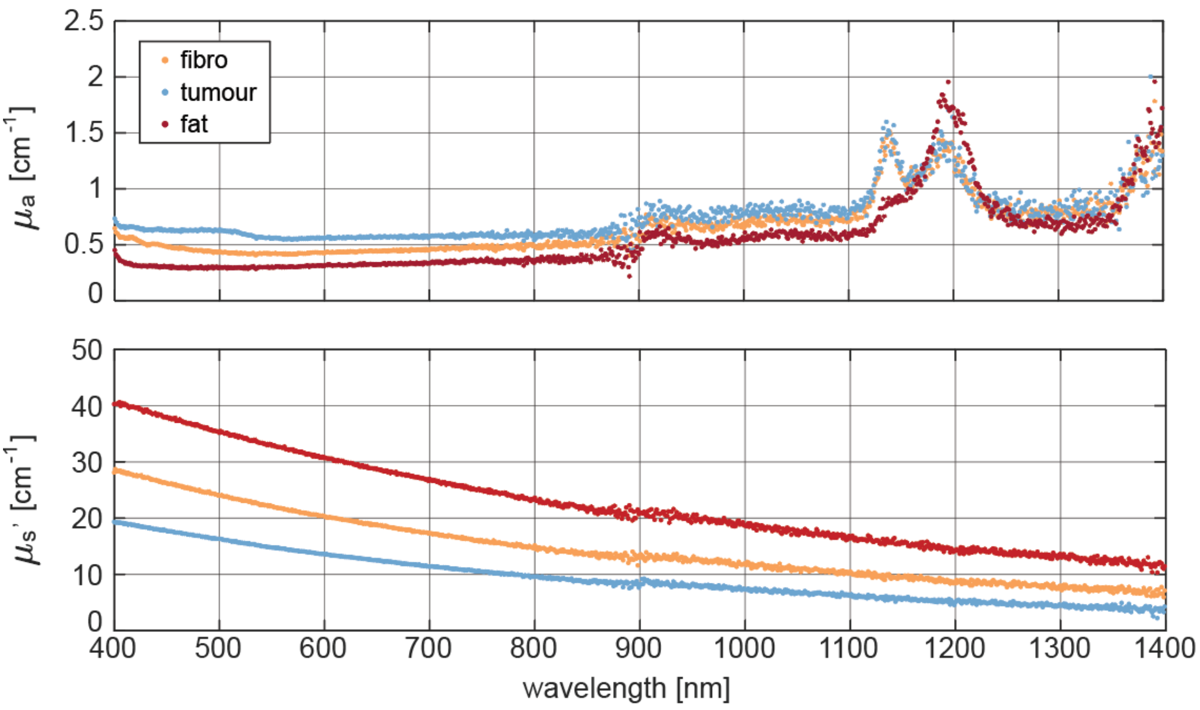


**Figure S3:** Optical absorption and reduced scattering coefficients for the three PVCP TMMs.

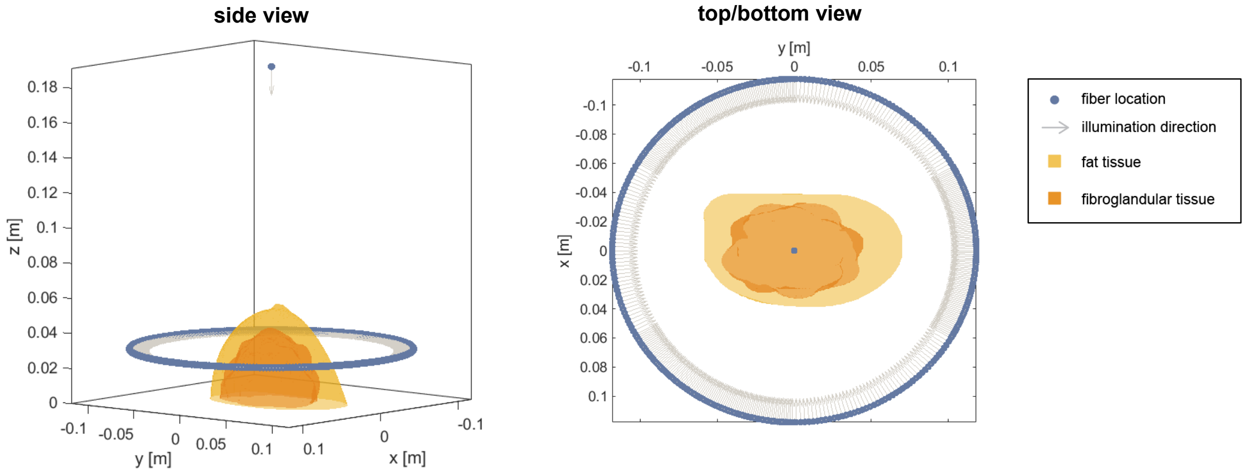


**Figure S4**: Side view and top/bottom view of the PAM2 illumination geometry as modelled in the Monte Carlo simulations.

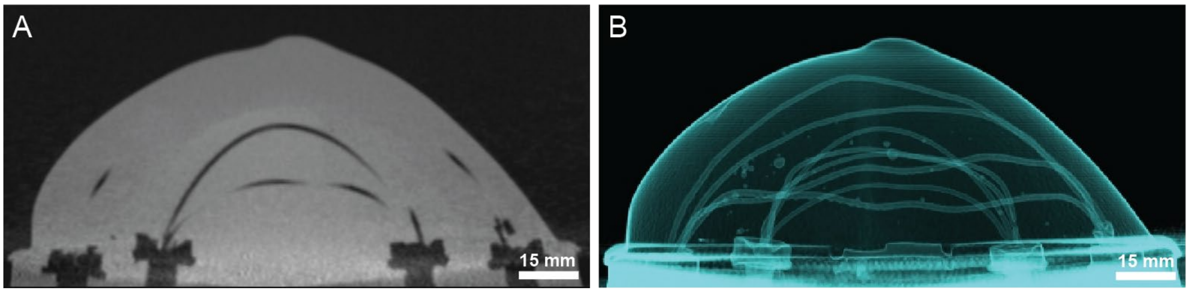


**Figure S5**: An enhanced CT-scan was made from the phantom with the ILUMA CT system (Imtec imaging LLC) with the tube current set to 3.8 mA. The acquired images have a 0.3 mm resolution. CT slice of the phantom is shown in (A). A projection view from the 3D scan with airways contrast (Radiant dicom viewer) is shown in (B). The channels and the connector cavities can be observed in both images. Connectors were removed before making the CT scan. A low contrast between the fat and fibroglandular layers exists with this imaging modality modality, but the channels show up with high contrast.

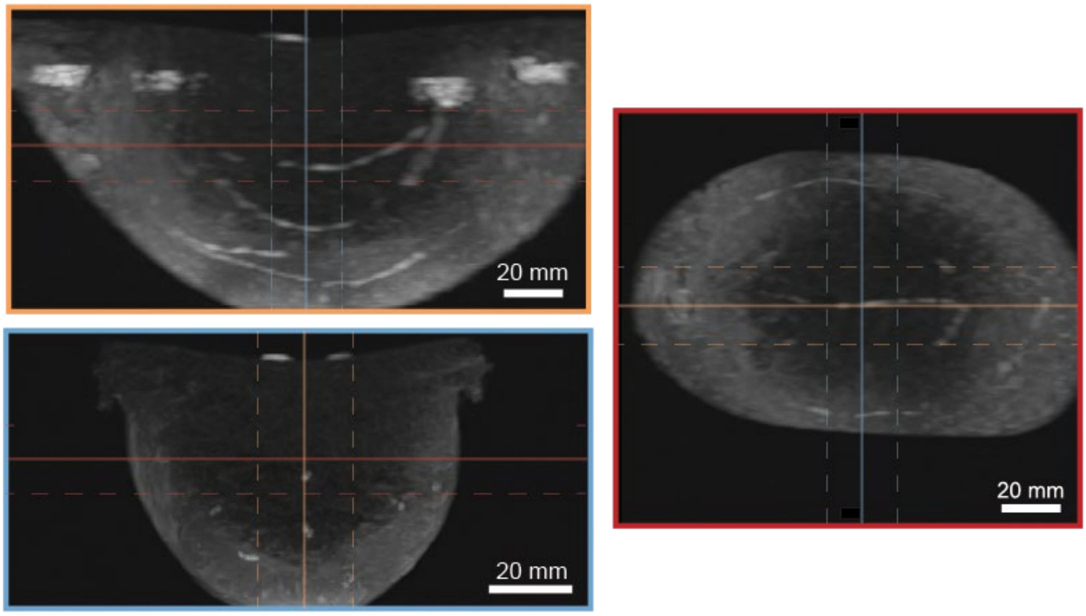


**Figure S6:** MRI images of the phantom were acquired with the 0.25 Tesla G-Scan Bio system (Esaote Benelux B.V.). Local maximum intensity projections (LMIP) along three perpendicular planes are shown here. Solid lines and dashed lines show region over which the LMIP were taken. Contrast between the fibroglandular and fat layers can be observed. The channels were filled with water and nicely show up in the images. The imaging resolution of this MRI is 0.5 mm, which is just enough to resolve the channels.
